# Mental health and tobacco use as predictors of breastfeeding duration among Virginia mothers

**DOI:** 10.1186/s12884-025-08464-5

**Published:** 2025-12-10

**Authors:** Elaine Cooper Russell, Kenneth W. Griffin, Amira A. Roess

**Affiliations:** Department of Global and Community Health, George Mason University, Fairfax, VA USA

**Keywords:** Breastfeeding, Lactation, Mental health, Depression, Anxiety, Tobacco use, Smoking, Comorbidities

## Abstract

**Background:**

Breastfeeding offers substantial positive health outcomes for both the mother and infant and has been identified as one of the most effective strategies for promoting infant health; however, the United States falls short in sustained breastfeeding rates. Additionally, with mental health conditions and substance use during pregnancy and the postpartum period affect nearly 1 in every 5 women in the U.S., more research is needed to better understand how these behavioral health factors, along with the co-occurrence of these factors, impact breastfeeding trends.

**Methods:**

Cross-sectional data from the Virginia Pregnancy Risk Assessment Monitoring System (PRAMS) from 2016 to 2022 were analyzed to assess how maternal mental health and tobacco use before, during, and after pregnancy was associated with breastfeeding duration in Virginia mothers (*n* = 5,704).

**Results:**

The simultaneous presence of depression and anxiety symptoms was negatively associated with breastfeeding duration; those who reported both depression and anxiety breastfed their infants nearly three weeks less (M = 10.45, SD = 7.82) than those who reported neither (M = 13.34, SD = 7.76, *p* < .001). The more timepoints a mother reported depressive symptoms (before, during, and after pregnancy) was associated with statistically significant decreases in average breastfeeding duration (*p* < .001). Mothers who reported smoking at any timepoint breastfed their babies nearly six weeks less (M = 7.63, SD = 7.49) than mothers who did not smoke (M = 13.4, SD = 7.71, *p* < .001). However, among smokers, statistically significant improvements in breastfeeding duration were found in those who took initiatives to quit smoking during pregnancy (*p* < .001). Receiving breastfeeding support from a lactation specialist was protective, especially in smokers, as those who met with a lactation consultant had 3.53 times the odds (95% CI: 2.30, 5.43) of breastfeeding at least 12 weeks. Marital status was also protective, as married women breastfed their infants more than a month longer (M = 14.22, SD = 7.11) than non-married women (M = 9.82, SD = 8.49). Additionally, smoking was associated with shorter breastfeeding duration for those with high anxiety (*p* = .03), particularly among non-Hispanic Whites, Medicaid enrollees, and mothers with a high school diploma or less.

**Conclusion:**

Enhanced smoking cessation, lactation support, and behavioral health services may be important for promoting breastfeeding among new mothers.

## Background

There are many short-term and long-term positive health outcomes of breastfeeding, for both the mother and the infant. For mothers, breastfeeding can reduce their risk of breast cancer, ovarian cancer, high blood pressure, and type 2 diabetes [1]. Breastmilk has antibodies that help foster a healthy immune system and protect infants from acute infectious illnesses [2]. Additionally, breastmilk can help lower an infant’s risk of obesity, diabetes, asthma, sudden infant death syndrome (SIDS), ear infections, stomach bugs, severe diarrhea, and respiratory tract infections [2, 3]. Longer duration of breastfeeding is associated with increased health protections [4]. The American Academy of Pediatrics (AAP) recommends exclusive breastfeeding for the first six months of life, followed by continued breastfeeding alongside the introduction of complementation foods for up to two years or longer [3]. Despite the many positive health effects of breastfeeding and the AAP recommendations, only about half (55.8%) of infants in the U.S. receive any breastmilk at six months, and only a quarter (24.9%) are exclusively breastfed until six months [5].

The prevalence and patterns of breastfeeding are shaped by numerous determinants. Economic and demographic factors, such as maternal age, income, education, material status, and race are associated with adherence to AAP’s breastfeeding guidelines [6, 7]. It has also been reported that poor maternal mental health and substance use is associated with suboptimal breastfeeding behavior [8, 9]. With mental health conditions and substance use disorders during pregnancy and the postpartum period affecting nearly 1 in every 5 women in the United States [10], it is important to better understand implications that poor behavioral health may have on breastfeeding duration.

It is estimated that 1 in every 10 women in the United States experience depression during pregnancy and 1 in every 5 experience anxiety [11, 12]. Elevated maternal anxiety and depression during pregnancy has been associated with preterm birth, low birthweight, miscarriage, pre-eclampsia, and developmental delays [12]. A systematic review from published literature from 2005 to 2016 found that postpartum depression can lead to an increase in risky behaviors, social and relationship problems, alcohol and drug use, and breastfeeding problems, along with poor cognitive functioning, emotional and behavioral problems, and medical disorders in the child [13, 14].

Although research links postpartum depression with lower breastfeeding initiation rates and shorter duration [8], a growing body of research has suggested that the relationship between postpartum depression and breastfeeding is bidirectional [15]. Breastfeeding releases hormones such as prolactin and oxytocin, which induces a state of calm and reduces stress in the breastfeeding mother [16], which can be beneficial for maternal mental health. Breastfeeding may be a protective factor against postpartum depression and can aid in recovery from depression symptoms [15]. This suggests that not engaging in breastfeeding early on may increase the risk of postpartum depression [15]. A longitudinal study reported that longer breastfeeding duration was related to a reduction in maternal depression and anxiety, and that those who stopped breastfeeding early were at an increased risk of poor mental health outcomes at six months [17]. However, more research is needed that analyzes maternal depression and anxiety separately from one another.

Maternal tobacco use is another concern for maternal and infant health. Using tobacco products (cigarettes or e-cigarettes) during pregnancy increases the risk of preterm birth, low birth weight, birth defects, and SIDS [18]. Tobacco use while breastfeeding poses several risks for infants as harmful chemicals can be passed to the infant through breastmilk [19]. Additionally, smoking tobacco products while breastfeeding exposes the child to secondhand smoke and may lower maternal milk supply [19]. Despite the negative consequences of maternal smoking on infant health, 5.4% of women in the United States report smoking during pregnancy, and a greater percent (7.2%) relapse to smoking in the postpartum period [20].

Research in the U.S. reports a significant negative relationship between maternal cigarette use and breastfeeding duration [18, 19]. A study published in 2006 that analyzed 2000–2001 Oregon Pregnancy Risk Assessment Monitoring System (PRAMS) data found that persistent smokers (mothers who smoked before, during, and after pregnancy) were 2.18 times more likely to not breastfeed at 10 weeks postpartum compared to nonsmokers [9]. A study conducted among Woman, Infant, and Children (WIC) recipients in Washington, DC found that mothers who smoked during the postpartum period breastfed their infants an average of 48.1 days less than mothers who did not smoke [21]. Studies conducted outside the U.S. have also reported that daily cigarette use was associated with shorter breastfeeding duration [22] and that that mothers who smoked during pregnancy had an increased odds of breastfeeding cessation prior to six months [23].

Using cross-sectional survey data collected by the Virginia Department of Health from 2016 to 2022, the primary focus of this paper is to analyze the association between mental health concerns (depression and anxiety) and tobacco use on breastfeeding duration in Virginia mothers. Additionally, this study examines differences across key demographic variables, such as race/ethnicity, age, health insurance type, marital status, and education, to investigate whether specific groups may be more or less likely to breastfeed. This study also analyzes the role that potential protective factors, such as lactation support and smoking cessation initiatives, may have on breastfeeding outcomes in at-risk groups. We hypothesize that the more timepoints a mother experiences a mental health concern or uses tobacco products, the lower the average breastfeeding duration. We also hypothesize that those who received breastfeeding information and support will experience enhanced breastfeeding outcomes.

While prior studies have focused on aspects of mental health and tobacco use in pregnant women or in the postpartum period, no recent studies conducted in the U.S. were found that examine these two behavioral components and their relationship with breastfeeding duration in one research study. This study analyzes patterns in breastfeeding duration based on maternal mental health (depression and anxiety) and tobacco use separately, and then combined. The methods used in this research are new to the field since it captures health information on women prior to, during, and after pregnancy rather than during one snapshot in time. Additionally, maternal anxiety during pregnancy is less studied, despite its high prevalence, and this research will help add to this body of literature.

## Methods

### Aim, design, and setting

The primary focus of this study is to analyze how patterns in breastfeeding duration may differ based on maternal mental health status (feelings of depression and anxiety) and cigarette use before, during, and after pregnancy. PRAMS is a population-based surveillance project between the Centers for Disease Control and Prevention (CDC) and state health departments throughout the United States. PRAMS was developed in 1987 and collects jurisdiction-specific, population-based data on maternal attitudes and experiences before, during, and shortly after pregnancy. It is the only surveillance system that provides data about pregnancy and the first few months after birth, and is representative of 81% of all live births in the U.S. For this study, researchers gained IRB approval from the Virginia Department of Health to use the PRAMS data specific to the Commonwealth of Virginia.

### Characteristics of participants and inclusion criteria

The study sample includes women who gave birth to a live infant between 2016 and 2022 and were selected to complete the Virginia PRAMS survey. Virginia birth certificates were used to randomly select 2,000 mothers each year to complete the survey. The sample is stratified by characteristics such as maternal age, race/ethnicity, geographic area of residence, and infant birth weight to allow for analysis of a wide range of public health disparities. Inclusion criteria for this research study included women aged 18 and older with infants aged 12 weeks or older at the time of survey completion (*n* = 5,704).

### Variables

#### Breastfeeding duration (outcome variable)

We created two breastfeeding duration variables, one continuous variable that indicates the number of weeks the infant was breastfed, and a dichotomous variable indicating whether the infant was breastfed for at least 12 weeks. Participants who indicated that they never breastfed were given a breastfeeding duration of 0 weeks. For mothers who indicated they were still breastfeeding at the time of survey completion, the age of their infant was used as their duration. Infants under 12 weeks old at the time of survey completion were excluded to avoid underestimating the breastfeeding duration. For example, it is unknown if a mother who is still breastfeeding their nine-week-old will still be breastfeeding at 12 weeks, thus, they were removed from the analyses.

#### Demographic variables

##### Mother’s age

Participants were asked to indicate today’s date (the day they completed the survey) along with their date of birth. Mother’s Age was calculated by subtracting today’s date from the mother’s birthday.

##### Mother’s education

Participants were asked to indicate their highest level of education achieved: 1) < = 8th grade, 2) 9th −12th grade, 3) High school grad/GED, 4) Some college/no degree/Associates, and 5) Bachelors/Masters/Doctorate. For analysis purposes, options 1–4 were combined and Mother’s Education was dichotomized into no degree (56.2%) and bachelor’s degree or higher (43.8%).

##### Mother’s race

Mothers were asked to indicate their race. Race was recoded to include: (1) White (55.4%), (2) Black (21.6%), (3) Asian (includes Chinese, Japanese, Filipino, and Other Asian) (5.1%), (4) Hispanic (15.1%), and (5) Other (includes American Indian, Hawaiian, Alaskan Native, Other, and Mixed Race) (2.8%).

##### Medicaid (infant)

Participants were asked to select the type of health insurance their infant is covered by at the time of survey completion. We created a dichotomous variable indicating whether (38.4%) or not (61.6%) the infant has Medicaid insurance.

##### Marital status

Participants were asked to indicate their marital status. Options included Married (65.9%) and Other (34.1%).

#### Mental health variables

##### Depression (pre-pregnancy)

Participants were asked to indicate (no or yes) if they had depression during the three months before their most recent pregnancy.

##### Depression (during pregnancy)

Participants were asked to indicate (no or yes) and if they had depression during their most recent pregnancy.

##### Anxiety (during pregnancy)

Participants were asked to indicate (no or yes) and if they had anxiety during their most recent pregnancy.

##### Postpartum depression

Mothers were asked: (1) how often they felt down, depressed, or hopeless, and (2) how often they have little interest or little pleasure in doing things you usually enjoyed since their infant was born. As recommended by the PRAMS codebook, if respondents answered “always” or “often” to either question, they were classified as experiencing postpartum depression.

##### Anxiety and depression scale

We created a depression and anxiety scale (0–3) that indicates the presence of no depression or anxiety at any timepoint, whether the mother had anxiety but not depression, had depression at any timepoint but not anxiety, or if the mother had both depression (at any timepoint) and anxiety during pregnancy. This scale isolates the presence of depression and anxiety from one another to analyze differences in breastfeeding duration based on the occurrence of one mental health condition compared to another.

##### Depression index

Since information about depression was collected pre, during, and post-pregnancy, we created a depression index variable which assessed the relationship between breastfeeding duration based on how many timepoints (0–3) the mother reported depressive symptoms.

#### Tobacco use variables

##### Smoked cigarettes (pre-pregnancy)

Participants were asked if they smoked any cigarettes in the past two years. For those who did smoke, they were asked to indicate how many cigarettes they smoked on an average day in the three months before they got pregnant. We created a dichotomous variable indicating whether the mother smoked cigarettes within the three months prior to getting pregnant.

##### Smoked cigarettes (during pregnancy)

Participants were asked to select the option that best indicates the number of cigarettes they smoked on an average day during the last three months of pregnancy. We created a dichotomous variable indicating whether the mother smoked cigarettes during the last trimester.

##### Smokes cigarettes (after pregnancy)

Participants were asked to select the option that best indicates the number of cigarettes they currently smoke on an average day. We created a dichotomous variable indicating whether the mother currently smokes cigarettes.

##### Smoking index

Since information about cigarette smoking was collected pre, during, and post-pregnancy, we created a smoking index variable which assessed the relationship between breastfeeding duration based on how many timepoints (0–3) the mother indicated they used cigarettes.

##### E-Cigarette use (pre-pregnancy)

Participants were asked if they used e-cigarettes in the past two years. For those who use e-cigarettes, they were asked to indicate how often they used these products on an average day in the three months before they got pregnant. We created a dichotomous variable indicating whether the mother used e-cigarettes during this time.

##### E-Cigarette use (during pregnancy)

Participants were asked to select the option that best indicates how often they used e-cigarettes during the last three months of their pregnancy. We created a dichotomous variable indicating whether the mother used e-cigarettes during this time.

#### Protective factors

##### Breastfeeding information

Mothers were asked to select if they received breastfeeding information from the following sources before or after their infant was born: (1) Mothers doctor, (2) Nurse, (3) Breastfeeding or lactation specialist, (4) Infant’s doctor, (5) Breastfeeding support group, (6) Breastfeeding hotline, (7) Family or friends, and (8) Other. We created two dichotomous variables, one indicating whether the mother received any breastfeeding information at all, and another variable indicating whether they receive information specifically from a lactation specialist.

##### Tried to quit smoking

Mothers who smoked in the three months prior to pregnancy were asked to indicate any initiatives they took to quit smoking. We created a dichotomous variable that indicates whether the mother took steps to quit smoking during pregnancy.

### Data analysis

All analyses were conducted in SPSS version 29. We calculated percentages of the total sample for each variable. Chi-square tests were conducted to compare the prevalence of breastfeeding at 12 weeks based on the various demographic, mental health, and tobacco use variables. Odds ratios with 95% confidence intervals were calculated to better understand the association between each predictor variable with breastfeeding at 12 weeks. Independent sample t-tests and ANOVA tests were run to compare the average breastfeeding duration (number of weeks) across the independent variables. Additionally, we ran a series of linear and logistic regression models to check for interactions among the behavioral health variables on breastfeeding duration, along with analyzing predictors of average breastfeeding duration.

The relationship between maternal mental health status and breastfeeding duration was analyzed in a variety of ways. First, researchers analyzed associations with breastfeeding at three months and length of breastfeeding duration (in number of weeks) by each timepoint and total number of timepoints the mother indicated they experienced a mental health concern. For instance, chi-square tests, t-tests, ANOVA tests, and odds ratios were calculated for maternal depression three months prior to pregnancy, during pregnancy, and postpartum depression, along with maternal anxiety during pregnancy. Additionally, we created a mental health scale to analyze differences in breastfeeding duration based on if a mother has no mental health concerns, has feelings of anxiety but not depression, depression but not anxiety, and both. We also analyzed how these patterns may differ between smokers and non-smokers.

Due to the potential for many significant findings as a result of the large sample size, odds ratios and confidence intervals were used instead of *p*-values for some analyses as a way to provide a more meaningful understanding of the magnitude of the relationships between variables, regardless of sample size. Additionally, we used the Bonferroni correction to avoid inflated Type I error rates when analyzing multiple comparisons.

## Results

### Sample overview

Table 1: *Characteristics of Study Participants* describes the demographic characteristics of the Virginia mothers included in this study. The sample was primarily non-Hispanic White (55.4%), married (65.9%), and had an average age of 30.59 (SD = 5.49). Less than half of the sample had a bachelor’s degree or higher (43.8%) and 38.4% had Medicaid insurance for their infant. One quarter (25.4%) of the sample reported experiencing depressive symptoms at some point within three months prior to getting pregnant to now, and 21.3% reported having anxiety during pregnancy. Nearly 12% of the sample reported cigarette use and 4% used e-cigarettes.

**Table 1:** Characteristics of study participants (*n* = 5704)

Characteristics	Study sample *n*(%)	Breastfed ≥ 12 weeks *n*(%)	OR (95% CI)	Average breastfeeding duration in weeks M(SD)	*P*-value
*Breastfed*	5116 (90.9%)	3824 (68.3%)	--	12.73 (7.90)	--
*Age*					
18–29	2339 (41.0%)	1316 (57.5%)	OR = 1 (ref)	11.23 (8.15)	*p* = < 0.001
30–50	3365 (59.0%)	2508 (75.7%)	OR = 2.31 (2.06, 2.59)	13.77 (7.53)	
*Education*					
No Degree	3181 (56.2%)	1722 (55.4%)	OR = 1 (ref)	11.01 (8.37)	*p* = < 0.001
Bachelors Degree+	2483 (43.8%)	2078 (84.7%)	OR = 4.48 (3.93, 5.11)	14.89 (6.60)	
*Race/Ethnicity*					
White (non-Hispanic)	3155 (55.4%)	2326 (74.9%)	OR = 1 (ref)	13.27 (7.27)	*p* = < 0.001
Black (non-Hispanic)	1228 (21.6%)	566 (47.1%)	OR = 0.30 (0.26, 0.34)	9.90 (8.78)	
Asian (non-Hispanic)	289 (5.1%)	231 (81.6%)	OR = 1.49 (1.09, 2.03)	15.51 (7.17)	
Hispanic	861 (15.1%)	581 (68.7%)	OR = 0.73 (0.62, 0.87)	13.76 (8.13)	
Other	162 (2.8%)	113 (72.9%)	OR = 0.90 (0.63, 1.30)	13.34 (7.09)	
*Medicaid (Baby)*					
Not on Medicaid	3476 (61.6%)	2741 (79.4%)	OR = 1 (ref)	14.08 (7.02)	*p* = < 0.001
Medicaid enrollee	2165 (38.4%)	1075 (50.3%)	OR = 0.26 (0.23, 0.30)	10.56 (8.68)	
*Marital Status*					
Not married	1946 (34.1%)	881 (46.5%)	OR = 1 (ref)	9.82 (8.49)	*p* = < 0.001
Married	3758 (65.9%)	2943 (79.4%)	OR = 4.44 (3.93, 5.0)	14.22 (7.11)	

### Breastfeeding overview

The majority of mothers who completed the Virginia PRAMS survey initiated breastfeeding (90.9%), and 68.3% breastfed their infant at least 12 weeks. Two-thirds of the sample indicated they were still breastfeeding at the time of survey completion (66.9%). Nearly all (97.1%) of the Virginia mothers said they received information about breastfeeding upon the birth of their child, with the most frequent sources coming from a lactation specialist (81.9%), the mother’s doctor (77.3%), or a nurse (76%). Those who received breastfeeding information breastfed their babies on average a month longer (M = 12.85, SD = 7.82) than those who did not receive such information (M = 8.83, SD = 9.1). Mothers who met with a lactation specialist to receive breastfeeding information upon the birth of their child had 2.65 (95% CI: 2.3, 3.05) times the odds of breastfeeding for at least three months compared to those who did not meet with a lactation consultant. This demonstrates the important role that health care professionals play in ensuring new mothers have the necessary breastfeeding information and support.

### Demographics

Mothers who were aged 30 and older tended to breastfeed their infants an average of two weeks longer (M = 13.77, SD = 7.53) than mothers under 30 (M = 11.23, SD = 8.15). Statistically significant increases in average breastfeeding duration were seen in mothers who were married and had higher education (p = < 0.001). On average, married women breastfed their babies more than a month longer (M = 14.22, SD = 7.11) than non-married women (M = 9.82, SD = 8.49), and those who have a bachelor’s degree or higher had 4.48 times the odds (95% CI: 3.93, 5.11) of breastfeeding for at least 12 weeks compared to mothers without a degree. Only 50.3% of babies whose health insurance was covered by Medicaid were breastfed for 12 weeks or longer, compared to 79.4% of babies not on Medicaid. Compared to White mothers, Asian mothers had 1.49 times the odds (95% CI: 1.09, 2.03) of breastfeeding for at least 12 weeks, whereas Black mothers had the lowest average breastfeeding duration among race groups (OR = 0.30, 95% CI: 0.26, 0.34). More demographics information can be found in Table 1.

### Mental health

Table 2: *Behavioral Health Predictors on Breastfeeding Duration* outlines key breastfeeding duration statistics based on whether the mother experienced a mental health condition or used tobacco products, separated by timepoint. One third of the sample (33%, *n* = 1,812) reported experiencing a mental health difficulty (anxiety during pregnancy and/or depression at any timepoint). Statistically significant (*p* = < 0.001) reductions in average breastfeeding duration were found with the presence of depression and anxiety. Depression was analyzed at three timepoints: three months prior to pregnancy, during pregnancy, and after pregnancy. Only 53% of mothers who felt depressed prior to getting pregnant breastfed for at least three months, compared to 70.6% of mothers who did not experience depression pre-pregnancy.

**Table 2:** Behavioral health predictors on breastfeeding duration

Characteristics	Study sample *n*(%)	Breastfed ≥ 12 weeks *n*(%)	OR (95% CI)	Average breastfeeding duration in weeks M(SD)	*P*-value
*Depression 3 months prior to pregnancy*					
Did not have depression	4906 (86.9%)	3412 (70.6%)	OR = 1 (ref)	13.13 (7.84)	*p* = < 0.001
Did have depression	740 (13.1%)	380 (53.3%)	OR = 0.47 (0.40, 0.56)	10.25 (7.84)	
*Depression last 3 months of pregnancy*					
Did not have depression	4802 (85.5%)	3342 (70.7%)	OR = 1 (ref)	13.13 (7.81)	*p* = < 0.001
Did have depression	812 (14.5%)	360 (52.9%)	OR = 0.47 (0.40, 0.54)	10.32 (8.10)	
*Anxiety last 3 months of pregnancy*					
Did not have anxiety	4420 (78.7%)	3079 (70.6%)	OR = 1 (ref)	13.12 (7.88)	*p* = < 0.001
Did have anxiety	1193 (21.3%)	678 (58.8%)	OR = 0.59 (0.52, 0.68)	11.25 (7.84)	
*Postpartum Depression*					
Does not have depression	4951 (87.9%)	3412 (69.8%)	OR = 1 (ref)	12.89 (7.80)	*p* = < 0.001
Does have depression	679 (12.1%)	379 (58.2%)	OR = 0.60 (0.51, 0.71)	11.67 (8.29)	
*Smoked cigarettes 3 months prior to pregnancy*					
Did not smoke	5015 (88.7%)	3568 (72.4%)	OR = 1 (ref)	13.39 (7.71)	*p* = < 0.001
Did smoke	637 (11.3%)	223 (35.8%)	OR = 0.21 (0.18, 0.25)	7.65 (7.51)	
*Smoked cigarettes last 3 months of pregnancy*					
Did not smoke	5418 (95.6%)	3728 (70.0%)	OR = 1 (ref)	13.03 (7.81)	*p* = < 0.001
Did smoke	247 (4.4%)	67 (27.9%)	OR = 0.17 (0.12, 0.22)	6.15 (7.05)	
*Smokes cigarettes now*					
Does not smoke	5269 (93.1%)	3700 (71.4%)	OR = 1 (ref)	13.24 (7.75)	*p* = < 0.001
Does smoke	392 (6.9%)	94 (24.7%)	OR = 0.13 (0.10, 0.17)	5.91 (6.71)	

**Fig. 1:**
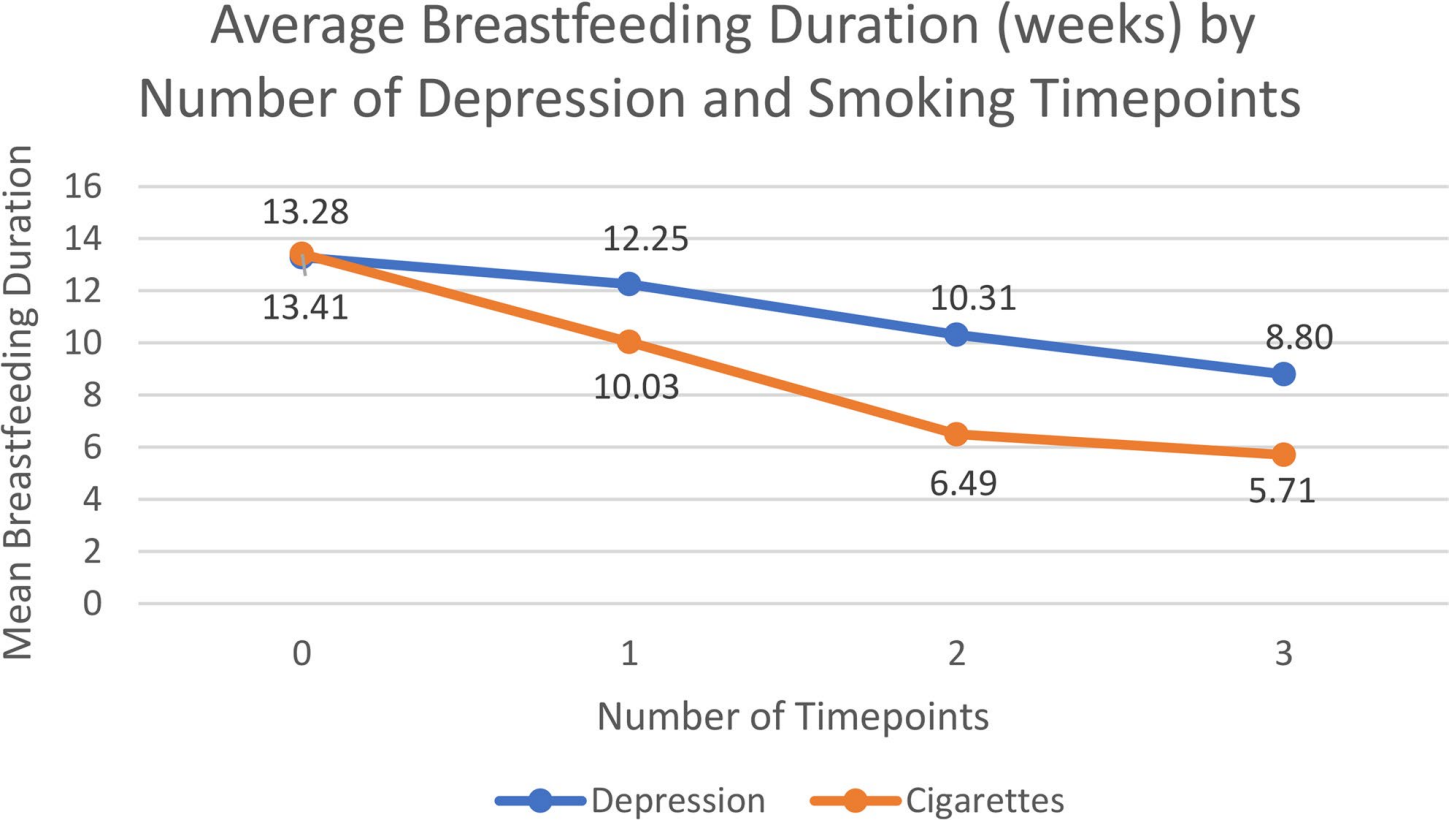
Average Breastfeeding Duration (weeks) by Number of Depression and Smoking Timepoints

Although the presence of depression at any timepoint was associated with a decreased odds of breastfeeding, the timepoint with the highest depression rate was during pregnancy (14.5%, *n* = 812). One in every five (21.8%. *n* = 1,217) mothers reported that a health care worker did not ask if they were feeling down or depressed during pregnancy. The number of timepoints a mother experienced depressive symptoms was inversely related to their average breastfeeding duration (Fig. 1). For example, those who did not experience depression at any timepoint had an average breastfeeding duration of 13.28 weeks (SD = 7.74), those with depression at one timepoint had an average of 12.25 weeks (SD = 8.14), two timepoints was 10.31 weeks (SD = 7.92), and those who felt depressed at all three timepoints had an average duration of 8.80 weeks (SD = 7.71).

**Fig. 2:**
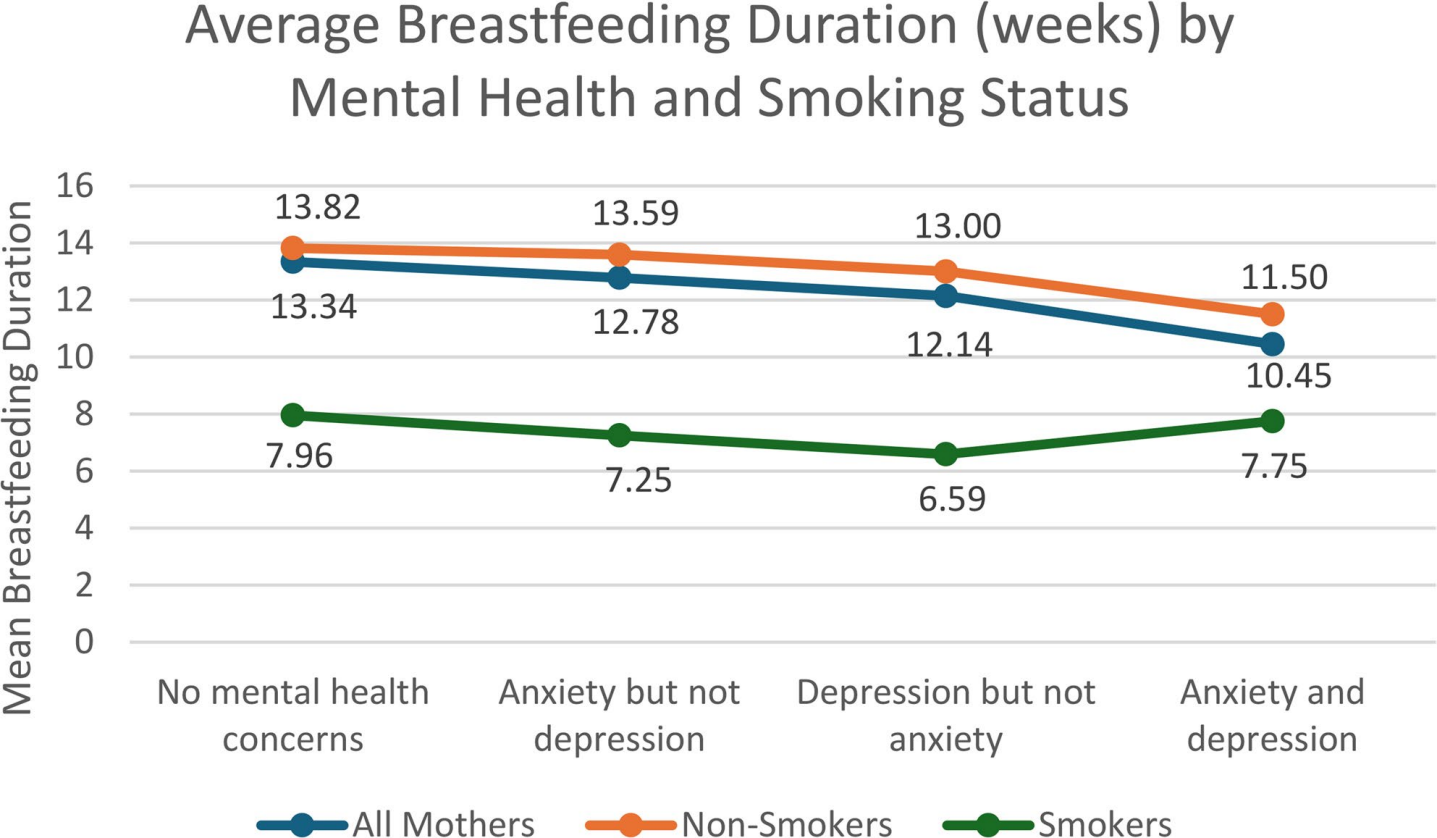
Average Breastfeeding Duration (weeks) by Mental Health and Smoking Status

Mothers who experienced anxiety during pregnancy breastfed their infants nearly two weeks less (M = 11.25, SD = 7.84) than mothers who did not experience anxiety during pregnancy (M = 13.12, SD = 7.88). However, when analyzing post-hoc results of an anxiety and depression scale, results indicated that compared to mothers with no mental health concerns, there were no statistically significant differences in breastfeeding duration for those who had only anxiety but no depression. Additionally, results indicated that there were no statistically significant differences in mean breastfeeding duration when comparing mothers who reported symptoms of anxiety but not depression to those who experienced depression but not anxiety. However, those who had both anxiety during pregnancy and depression at any timepoint had a significantly lower breastfeeding duration than any other group (Fig. 2). This indicates that the presence of anxiety alone may not have a drastic effect on breastfeeding duration; however, the relationship with breastfeeding duration may be altered by the co-occurrence of both.

To analyze this relationship further, we created an interaction term to test for moderation. A significant interaction was found between depression at any stage and anxiety during pregnancy (*p* =.045). To visualize and better understand patterns in the relationship, we plotted the interaction between anxiety and depression on breastfeeding duration. Mothers who experienced both anxiety and depression showed larger decreases in average breastfeeding duration compared to those who experienced only anxiety or only depression. This indicates that anxiety does not seem to have as strong of an effect unless depression is present, and that the relationship between anxiety and breastfeeding is moderated by depression.

### Tobacco use

#### Cigarette smoking

Approximately one in every nine participants (11.7%, *n* = 665) in this study reported smoking, and more than half (57.8%, *n* = 366) of mothers who smoked pre-pregnancy continued to smoke after having their baby. Smoking cigarettes at any timepoint was negatively associated with sustained breastfeeding duration. Only 35.8% of mothers who smoked cigarettes within the three months prior to getting pregnant breastfed their babies for 12 weeks or longer, compared to 72.4% of mothers who did not smoke. Mothers who smoked cigarettes during pregnancy breastfed their babies nearly seven weeks less (M = 6.15 weeks, SD = 7.05) than mothers who did not use cigarettes during this time period (M = 13.03 weeks, SD = 7.81). However, an even lower average breastfeeding duration was seen in mothers who indicated they currently smoke upon taking the PRAMS survey; non-smokers breastfed their babies an average of 13.24 weeks (SD = 7.75) compared to only 5.91 weeks (SD = 6.71) for mothers who smoked in the early postpartum period. More information about breastfeeding duration based on the timepoint in which the mother used tobacco products can be found in Table 2.

Additionally, the more timepoints the mother smoked, the more pronounced the decrease in mean breastfeeding duration. For instance, a mother who only reported smoking at one timepoint had an average breastfeeding duration of 10.03 weeks (SD = 7.83), compared to an average of 5.71 weeks for those who smoked at all three timepoints (SD = 6.81) (Fig. 1).

Of the mothers who reported smoking in the three months leading up to pregnancy, 81.3% (*n* = 565) tried to quit smoking once they became pregnant. Mothers who took initiatives to quit smoking breastfed their babies approximately three weeks longer (M = 8.19, SD = 7.68) than those who did not take initiatives to quit smoking (M = 5.09, SD = 6.45). The most frequently reported techniques to help mothers quit smoking included setting a specific date to stop smoking (*n* = 221, 31.9%), using nicotine patches (*n* = 56, 8.1%), and using educational resources (booklets, videos, or other materials) to help (*n* = 40, 5.8%).

Receiving breastfeeding support from a lactation specialist helped drastically improve overall breastfeeding duration, especially in smokers. Smokers who met with a lactation consultant breastfed their babies an average of five weeks longer (M = 9.12, SD = 7.23) compared to those who did not meet with a specialist (M = 3.98, SD = 6.96).

#### E-cigarettes

Nearly 4% (*n* = 215) of participants used e-cigarettes within three months before getting pregnant and less than 1% used them during pregnancy (*n* = 51). Mothers who used e-cigarettes had a statistically significant (*p* = < 0.001) decrease in average breastfeeding duration compared to mothers who did not use e-cigarettes, but the decrease was not as detrimental as regular cigarettes. Those who used e-cigarettes during pregnancy breastfed their babies an average of 5.74 weeks shorter than those who did not use e-cigarettes.

### Mental health and tobacco use

Experiencing symptoms of anxiety during pregnancy (40.8%, *n* = 271) and depression at any timepoint (45.4%, *n* = 302) were both twice as prevalent in smokers compared to non-smokers (18.6%, *n* = 918; 22.7%, *n* = 1,133). A significant interaction was found for the relationship between anxiety during pregnancy and cigarette smoking at any timepoint on the average number of weeks the mother breastfed (*p* =.034). This means that the effect of smoking differs for mothers who experienced anxiety compared to mothers who did not have anxiety. Plotting the interaction revealed that smoking has a stronger negative effect on breastfeeding duration for mothers with high anxiety. This means that while smoking might reduce breastfeeding duration for all mothers, the reduction is greater for mothers who are also highly anxious.

The interaction still remained significant when adjusting for relevant demographic variables, such as age of the mother, education, race, Medicaid status, and marital status (*p* =.044). To analyze this relationship further, we split the dataset to observe any differences that may persist among demographic groups. The interaction was significant for Whites (*p* =.033), those with Medicaid insurance (*p* =.044), and mothers without a college degree (*p* =.035).

Next, we split the dataset by smoking status to further analyze potential differences in average breastfeeding duration by mental health status and tobacco use (Fig. 2). ANOVA test results indicated that the presence of a mental health condition was statistically significant in non-smokers (*p* = < 0.001), but not in smokers (*p* =.50). Regardless of the presence of a mental health concern, those who smoked had statistically significant reductions in breastfeeding duration. Post-hoc results revealed that in non-smokers, there was no statistically significant difference in average breastfeeding duration for mothers who reported only depressive symptoms or only anxiety; however, those who reported both depression and anxiety had a statistically significant lower average breastfeeding duration (*p* = < 0.001).

We conducted a series of multiple linear regression analyses to examine predictors of average breastfeeding duration (Table 3: *Multiple Linear Regression Analysis of Breastfeeding Duration by Smoking Status*). Due to large differences observed in smokers and non-smokers, we analyzed these two groups separately. In the non-smoker group, factors such as higher education, older age, being married, and receiving support from a lactation specialist were associated with statistically significant increases in average breastfeeding duration, while being Non-Hispanic Black and having mental health difficulties were associated with a lower average duration. In the smoker group, being married, taking initiatives to quit smoking, and receiving support from a lactation specialist were associated with statistically significant increases in average breastfeeding duration, while decreased duration was observed in Non-Hispanic Black participants.

**Table 3:** Multiple linear regression analysis of breastfeeding duration by smoking status

Characteristic	Non-Smokers			Smokers		
	Standardized Beta	t	*P*-value	Standardized Beta	t	*P*-value
Medicaid Status	0.02	0.99	0.32	− 0.07	−1.56	0.12
Race (Non-Hispanic Black vs. Other)	− 0.07	−4.49	< 0.001**	− 0.09	−2.17	0.03*
Education	0.11	6.04	< 0.001**	0.06	1.31	0.19
Age	0.04	2.54	0.01*	− 0.02	− 0.39	0.70
Marital Status	0.12	6.24	< 0.001**	0.13	2.82	0.005*
Mental Health	− 0.05	−3.45	< 0.001**	− 0.03	− 0.70	0.49
Lactation Specialist	0.12	8.38	< 0.001**	0.29	7.35	< 0.001**
Tried to Quit Smoking	--	--	--	0.09	2.20	0.029*

** *p* <.001, * *p* <.05

## Discussion

The purpose of this study was to provide a better understanding of how maternal mental health and tobacco use - before, during, and after pregnancy - is associated with breastfeeding duration in Virginia mothers. This study analyzed how breastfeeding trends at various timepoints may change based on the type of mental health difficulties encountered and tobacco use, along with how breastfeeding rates may differ by relevant demographic factors. Additionally, this study can help the field better understand how certain comorbidities and modifiable risk factors may affect breastfeeding success.

This study analyzed trends in breastfeeding duration when the presence of depression and anxiety were isolated from one another, and then combined. Our study found that mothers who reported both anxiety and depression at any timepoint had a significantly lower breastfeeding duration than either condition alone, which is in line with other studies [24]. Very few other studies have analyzed the presence of depression and anxiety separately in its relationship to breastfeeding duration. This is the first known U.S. study that found depression as a moderator in the relationship between anxiety and breastfeeding duration, which demonstrates that the relationship between anxiety and breastfeeding duration may be stronger with the presence of depression. For instance, depression may amplify the breastfeeding challenges that mothers with anxiety may face, thus leading to earlier breastfeeding cessation. It is important to detect and treat maternal mental health concerns prior to and during pregnancy to avoid harmful health behaviors and concerns in the postpartum period.

Findings from this study indicate that the presence of a mental health condition reduced overall breastfeeding duration in non-smokers, but not in smokers; mothers who smoked had significantly lower breastfeeding rates, regardless of mental health status. This could be due to the highly comorbid relationship between mental health and tobacco use [25]. For example, more smokers may also experience a mental health concern and use smoking as a coping mechanism, thus the negative impact of depression and/or anxiety may not be as noticeable. Further, this could also explain the significant interaction between anxiety and smoking on the relationship with breastfeeding duration. The presence of anxiety alone did not drastically impact breastfeeding duration; however, smoking appears to have a stronger negative effect on breastfeeding duration for mothers who have anxiety.

Our findings revealed that only a third of women who smoked at any timepoint breastfed for 12 weeks or longer, and the more timepoints a mother smoked, the lower their average breastfeeding duration. These findings are consistent with a 2017 study [26] which found that among former smokers who initiated breastfeeding, many weaned prior to two months. However, the same study [26] found that the majority of women who quit smoking during pregnancy had higher breastfeeding rates, which is consistent with our results that revealed that the intent to quit smoking during pregnancy is associated with greater breastfeeding outcomes. A 2021 study conducted in low-income women also reported that mothers who stopped smoking during pregnancy had a 50% greater likelihood of breastfeeding initiation compared to mothers who continued smoking [21]. Further, analyses from the 2000–2001 Oregon PRAMS data suggested that mothers who quit smoking during pregnancy did not have a statistically significant higher risk for early weaning compared to nonsmokers [9].

This study found that shorter breastfeeding duration outcomes were found in mothers who are younger, less educated, and non-married, which is consistent with prior literature [27]. Higher educational attainment is associated with engagement in more positive health behaviors, greater social interactions, and a higher income [28]. Education and marriage may play a role in longer breastfeeding duration by providing mothers with greater access to information, support, and resources [29].

This research identified certain groups who could benefit from focused interventions and enhanced breastfeeding support. While meeting with a lactation specialist was associated with longer breastfeeding duration, this difference was especially notable in smokers. Although greater strides should be made to help mothers quit smoking, it is essential to also ensure they receive adequate lactation support. Donath et al. [30] conducted a study on pregnant women in the United Kingdom to analyze breastfeeding duration based on the mother’s intent to breastfeed and smoking status, and found that breastfeeding rates among smokers are lower primarily due to lack of motivation to breastfeed, rather than the effect of smoking on milk supply. Health care providers can play an important role in helping improve breastfeeding duration outcomes by ensuring they are screening mothers for mental health concerns and tobacco use, providing treatment and information where appropriate, along with providing adequate information and support to boost overall confidence and motivation to breastfeed.

A limitation of this study is that long-term breastfeeding duration could not be assessed, since the survey was administered only 2–4 months after delivery. Due to the cross-sectional nature of the study, there is potential for recall bias when asked questions about before and during pregnancy, and mothers may under or over report tobacco use or feelings of mental health concerns. Information related to anxiety was only asked during pregnancy, so relationships with breastfeeding duration and anxiety pre and post pregnancy could not be examined. Mental health status was assessed through maternal self-report based on the PRAMS questionnaire and may not necessarily indicate a clinical diagnosis. The data used for this study was in Virginia mothers who recently gave birth, and may not be generalizable to the United States as a whole. Additionally, this survey lacked information on maternal employment status; future studies should assess how the mothers’ employment status may impact the relationship between behavioral health factors and breastfeeding duration.

This present study has several strengths. To our knowledge, this is the first U.S. study that examines the relationship between symptoms of depression, anxiety, and tobacco use at different timepoints on breastfeeding duration outcomes in one study. Second, this research adds to the limited body of literature that analyzes associations with maternal anxiety on breastfeeding duration. Additional strengths include the large sample size that is representative of approximately 81% of live births in the state and study design. The use of birth certificates was an effective way to identify the study population of focus, and the sampling strategy is stratified by infant birth weight to ensure an adequate representation of health disparities. The dataset also included a wide variety of demographic and health variables to analyze.

With low rates of sustained breastfeeding in the United States and increased instances and co-occurrence of maternal mental health concerns and tobacco use [2, 10], the findings from this study are important to help better understand the relationship between behavioral health factors and breastfeeding duration in new mothers. These findings are important in helping identify behavioral risk factors, as well as demographic groups that may be at risk of lower breastfeeding rates. Additionally, these findings can provide insights to inform future interventions or public health initiatives to help support breastfeeding and enhance maternal mental health.

## Conclusion

The findings from this study highlight the important role that health care professionals, especially lactation specialists, physicians (both the mother’s and the infant’s doctor), and nurses play in helping mothers initiate and prolong breastfeeding by ensuring that mothers receive adequate information about breastfeeding during pregnancy and immediately upon birth. Additionally, health care professionals can play a vital role in screening for mental health and substance use during pregnancy, along with helping mothers receive the necessary treatment and care. Enhanced resources and interventions are needed to help more pregnant women quit smoking, and continue to remain tobacco-free after birth.

## Data Availability

The data used in this study were obtained from the Virginia Department of Health. Due to privacy restrictions, these data are not publicly available. Researchers interested in obtaining Virginia PRAMS data must submit a data request to the Virginia Department of Health, following their established application procedures, which are outlined here:https://redcap.vdh.virginia.gov/redcap/surveys/?s=4PP8XDLR3D. General information about PRAMS data access can be found on the CDC PRAMS website: https://www.cdc.gov/prams/php/data-research/index.html. The authors did not have special access privileges beyond those outlined in the standard application procedures.
